# Study on the Influence of Mining Activities on the Quality of Deep Karst Groundwater Based on Multivariate Statistical Analysis and Hydrochemical Analysis

**DOI:** 10.3390/ijerph192417042

**Published:** 2022-12-19

**Authors:** Chen Li, Herong Gui, Yan Guo, Jiayu Chen, Jun Li, Jiying Xu, Hao Yu

**Affiliations:** 1 School of Earth and Environment, Anhui University of Science and Technology, Huainan 232001, China; 2 National Engineering Research Center of Coal Mine Water Hazard Controlling, Suzhou University, Suzhou 234000, China; 3 School of Resources and Civil Engineering, Suzhou University, Suzhou 234000, China

**Keywords:** karst groundwater, mining disturbance, hydrogeochemical evolution, fuzzy comprehensive evaluation, principal component analysis

## Abstract

Long-term mining activities have changed the hydrogeochemical evolution process of groundwater and threatened the safe use of groundwater. By using the methods of hydrochemistry and multivariate statistical analysis, this study determined the hydrogeochemical evolution mechanism affecting the quality of karst groundwater by analyzing the conventional hydrochemistry data of the karst groundwater of the Carboniferous Taiyuan Formation in Hengyuan Coal Mine in the recent 12 years. The results show that, under the disturbance of mining, the quality of karst groundwater in Taiyuan Formation is poor, mainly because the contents of Na^+^+K^+^ and SO_4_^2−^ are too high to allow usage as drinking water. The reason for the high content of SO_4_^2−^ in karst groundwater lies in the dissolution of gypsum and the oxidation of pyrite, and the high content of Na^+^+K^+^ lies in the cation exchange. Influenced by the stratum grouting, the circulation of karst groundwater is improved, the cation exchange is weakened, and the desulfurization is enhanced. In the future, it is predicted that the hydrochemical type of karst groundwater in Taiyuan Formation in the study area will evolve from SO_4_-Ca·Mg type to HCO_3_-Ca Mg type.

## 1. Introduction

Karst area accounts for about 15.2% of the global land area, and is widely distributed in China [1], the United States [2], South Africa [3], Ireland [4], and many other countries in the world. About 25% of the world population depends on water from karst aquifers due to the large volume of water stored in these formations and excellent water quality [5]. In China, karst groundwater is usually stored in Carboniferous-Permian strata with abundant coal resources [6].

**Figure 1:**
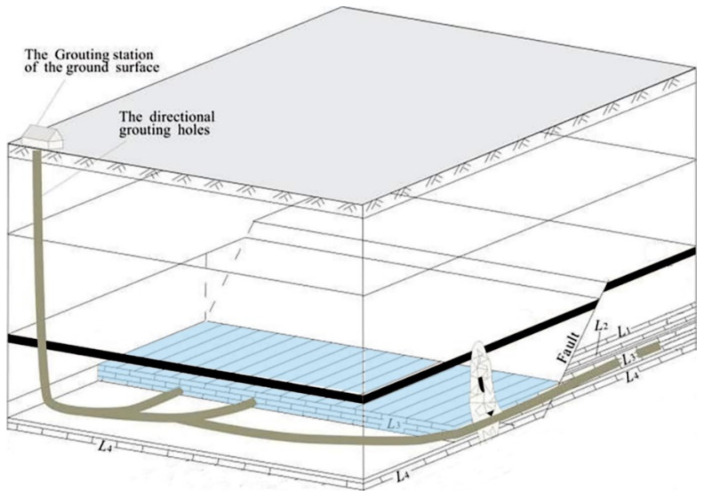
Three-dimensional model diagram of high-pressure grouting technology with directional drilling on the ground.

Coal mining causes fractures in underground rock stratum, greatly change the permeability of surrounding rock, and a large amount of groundwater will flow into the mine along the water conducting fissure, thus easily leading to water inrush disasters [7]. Water inrush accidents cause economic loss and potentially injuries or even death. According to preliminary statistics, about 47.5% of coal mines in China are affected by karst underground water disasters, and Huaibei coalfield is one of the most seriously threatened mining areas in China by karst water disasters [8]. The mine water inrush is mainly related to the water pressure acting on the floor water resisting layer and the thickness of the floor water resisting layer. Therefore, drainage to reduce the water pressure and grouting to increase the floor thickness are two effective methods to prevent water inrush accidents. Due to the uncertainty of the safety factor and the serious damage to the balance of the groundwater system, the method of reducing the water pressure by drainage is rarely used. At present, the high-pressure grouting technology of surface directional drilling is widely used in Huaibei Coalfield (Figure 1) to reinforce the third layer of thin limestone (L_3_) of Carboniferous Taiyuan Formation. While increasing the thickness of the floor aquifers, it effectively blocks the karst fractures and water diversion channels (such as faults) that pass through the third layer of thin limestone, to prevent the occurrence of karst groundwater water inrush disasters [9].

In recent years, the shallow easy-to-mine coal resources have been exhausted, and the development intensity of deep coal resources is gradually increasing [10]. On the one hand, with the in-depth exploitation of coal resources under conditions of high-water pressure and high-geostress, the threat of karst groundwater water inrush disasters increases [11]. On the other hand, the large-scale grouting reinforcement project to prevent water inrush disasters has caused a series of problems, such as the change of groundwater runoff conditions, groundwater acidification, and water quality exceeds the standard for drinking water [12]. These artificial disturbances have brought great challenges to the safe coal mining in the mining area and the production, life and health of the surrounding residents. Therefore, mine water inrush and water pollution are two groundwater problems that need to be solved urgently in China’s mining areas [13]. It is the theoretical basis for the prevention and control of karst water disasters and groundwater pollution to clarify the hydrogeochemical characteristics of groundwater and scientifically evaluate the quality of groundwater.

Conventional hydrochemistry, multivariate statistical analysis, and other methods have been widely used in the study of hydrogeochemical evolution in mining areas in recent years [14]. Especially, some scholars have used these methods in groundwater research to determine the influence of mining activities on the groundwater quality in the process of groundwater circulation, which enhances our understanding of the natural and human processes that control the hydrogeochemical evolution of groundwater in mining areas [15]. For example, Li et al. [16] based on the fuzzy set theory to make a comprehensive evaluation of the groundwater environment in the mining area. Wang et al. [17] used the improved fuzzy comprehensive evaluation method to scientifically evaluate the quality of karst groundwater in the mining area, which provided to be a useful reference for the rational utilization of local groundwater resources. These studies provide a theoretical basis for the development of groundwater quality management and hydrochemical prediction of environmental pollution control, and show that coal mine production has a great influence on chemical evolution and water quality of groundwater in mining areas.

The hydrogeochemical evolution characteristics are formed by the long-term interaction between groundwater and the surrounding environment [18]. Therefore, hydrogeologic exploration should also be a long-term continuous process. However, the karst groundwater in Carboniferous Taiyuan Formation is buried deep, and it is difficult to obtain samples. Few researchers have studied the hydrogeochemical evolution and water quality change of Taiyuan Formation karst groundwater in a long-term scale. In this study, Hengyuan Coal Mine, a typical coal mine in Huaibei Coalfield, China, is taken as the research area, and the conventional hydrogeochemical major ions (Na^+^, K^+^, Ca^2+^, Mg^2+^, Cl^−^, HCO_3_^−^, and SO_4_^2−^) data of karst groundwater in Taiyuan Formation in recent 12 years are used to: (1) evaluate the change of karst groundwater quality in the study area in a long-term scale; (2) determine the hydrochemical process that has influenced the groundwater quality in the mining area for a long time, and analyze the source and formation mechanism of major ions in groundwater; and (3) make a reasonable prediction on the hydrochemical characteristics of karst groundwater in Taiyuan Formation in the future. The research results are helpful to better understand the hydrogeochemical system of coal mining areas and provide reference for safe production and utilization and protection of deep karst groundwater resources in Huaibei coalfield.

## 2. Study Area

**Figure 2:**
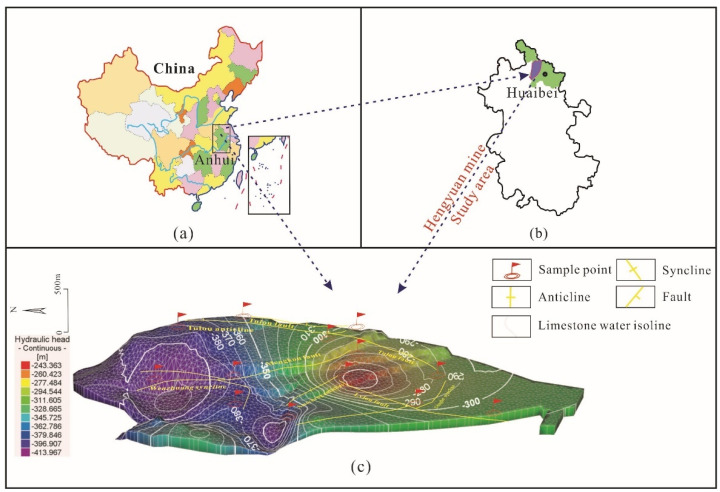
Geographical location of the Hengyuan Coal Mine. (**a**) Anhui Province in China, (**b**) the terrain of Anhui Province and the study area in Anhui Province, (**c**) geological map of the study area and location of sampling points.

Hengyuan Coal Mine is located in Huaibei City in the north of Anhui Province, China (Figure 2). Its geographical coordinates are 116°37′30″~116°41′15″ E and 33°54′30″~33°58′00″ N. The climate in this region is mild, belonging to temperate semi-humid monsoon climate. According to the local meteorological observation data, the annual average temperature in this area is 16.8 °C, the annual average rainfall is 1067 mm, and the rainfall is mainly concentrated in July and August each year.

**Figure 3:**
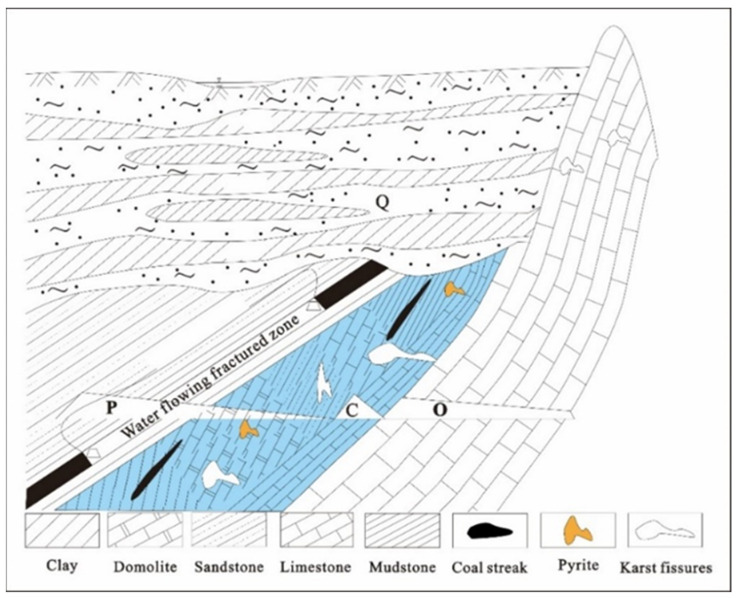
Hydrogeological profile of the study area.

The study area is flat, with no exposed bedrock, and is covered by thick Cenozoic loose layer. Carboniferous (C) Taiyuan Formation limestone is located in the lower part of Permian (P) coal measure stratum, and consists of limestone, sandy shale, and mudstone sandwiched with several thin coal seams (Figure 3). Geological exploration data show that carbonate minerals (dolomite, calcite) are the mainly found in the limestone strata of Taiyuan Formation in the study area, which contain thin mudstone and coal lines, and pyrite nodules are also found. Karst groundwater mainly stores in limestone karst fissures, and continuously migrates and enriches chemical components with these rock minerals in the process of circulation. Therefore, the formation and evolution of karst groundwater hydrogeochemical characteristics are affected by the formation lithology in the study area.

## 3. Sampling Test and Research Method

### 3.1. Sampling and Testing

From 2007 to 2022, a total of 61 Taiyuan Formation karst groundwater samples were collected from Hengyuan Coal Mine, including 35 samples from coal mining period before grouting (2007–2013), 15 samples from grouting period (2018–2020) and 11 samples from coal mining period after grouting (2021–2022). These samples were taken from drainage holes and hydrological observation holes in the mining area (Figure 2c).

Before sampling, the 2.5 L sampling bucket was rinsed three times with deionized water and washed three times with the water sample to be sampled. After sample collection, the portable instrument OHAUS (Shanghai, China) was used to test the pH, TDS and other indicators of water samples on site. All samples were sent to the laboratory within 8 h and stored in a refrigerator at 4 °C until further testing. Before the test, the water samples were filtered by a 0.45 μm filter membrane.

The contents of Na^+^, K^+^, Ca^2+^, Mg^2+^, Cl^−^ and SO_4_^2−^ in the water were measured by an ion chromatograph (ICS-600-900), and the contents of HCO_3_^−^ and CO_3_^2−^ were determined by titration with acid-base standard solution. The error of anion and cation balance of the measured data was within the standard limit of 5%, which indicated that the test results were reliable.

SPSS 27.0 software was used to analyze the correlation of conventional ion data, and the hydrochemistry simulation software PHREEQC 3.7.0 was used to calculate the mineral saturation index of karst groundwater, and the software Origin 2022 and CorelDraw 2018 were used to draw Piper diagram, Gibbs diagram, ion proportion coefficient diagram, and principal component analysis diagram, etc.

### 3.2. Research Methods

#### 3.2.1. Principal Component Analysis (PCA)

Principal component analysis is a multivariate statistical method that uses the idea of dimension reduction to transform multiple indicators into a few unrelated comprehensive indicators on the premise of little information loss [19]. Its essence is to seek the comprehensive substitute object of related variables through the correlation of original variables and ensure the minimum information loss in the process of transformation [20]. In this study, Principal component analysis is used to explore the main factors affecting the hydrogeochemical process of karst groundwater of Taiyuan Formation in the study area. The main steps are as follows [21]:

1. The data of hydrochemical indexes was standardized to eliminate the errors caused by different dimensions;
2. The correlation coefficient was calculated according to the standardized data matrix, and the eigenvalues and eigenvectors of the correlation coefficient matrix were calculated;
3. The principal components were determined, the variance contribution rate and cumulative variance contribution rate of the data set were calculated, and professional explanations were given to each principal component according to the actual situation.

#### 3.2.2. Fuzzy Comprehensive Evaluation

Groundwater is affected by many factors in the process of circulation, which makes the classification of water quality levels fuzzy [22]. The fuzzy comprehensive evaluation applies the fuzzy relation comprehensive principle to deal with the “fuzzy” phenomenon, quantifies some uncertain factors through the degree of membership, and then makes a comprehensive evaluation, to make intuitive judgement on uncertain factors [23]. However, when the pollution of a single factor is serious, the traditional fuzzy comprehensive evaluation results will be affected to some extent [16]. Therefore, this study adopts the improved fuzzy comprehensive evaluation method to evaluate the water quality of karst groundwater in Taiyuan Formation. The specific steps are as follows:

1. Establish evaluation factor set and evaluation language set

Establish the evaluation factor set K = {K_1_, K_2_,... K_n_} according to the selected evaluation factors. According to the water quality category specified in the Groundwater Quality Standard (GB/T14848-2017) in China [24], the evaluation corpus L = {I, II, III, IV, V} is established. Groundwater quality grading standards are shown in Table 1.

2. Establish fuzzy relation matrix

Since the higher the water quality grade of the conventional water quality index is, the lower its standard value is, and the subordination degree of the water quality is linearly distributed, the “reduced half trapezoidal stepwise method” is generally adopted [25]. The calculation formula of subordination degree of water quality at all levels is as follows:

Class I:

(1) $$a_{i1}=\{\begin{array}{c} 1\;\;\;\;t_{i}\leq g_{i1}\\ \frac{g_{i2}-t_{i}}{g_{i2}-g_{i1}}\;g_{i1}{<t}_{i}{<g}_{i2}\\ 0\;\;\;\;t_{i}{>g}_{i1} \end{array}$$

For class II~IV:

(2) $$a_{\mathrm{ij}}=\{\begin{array}{c} 1-\frac{g_{\mathrm{ij}}-t_{i}}{g_{\mathrm{ij}}-g_{\mathrm{ij}-1}}\;\;\;\;\;g_{\mathrm{ij}-1}\leq t_{i}\leq g_{\mathrm{ij}}\\ 0\;\;\;\;\;\;\;t_{i}\leq g_{\mathrm{ij}-1}{,t}_{i}{>g}_{\mathrm{ij}+1}\\ \frac{g_{\mathrm{ij}+1}-t_{i}}{g_{\mathrm{ij}+1}-g_{\mathrm{ij}}}\;\;\;\;\;\;g_{\mathrm{ij}}{<t}_{i}{<g}_{\mathrm{ij}+1} \end{array}$$

Class V:

(3) $$a_{i5}=\{\begin{array}{c} 0\;\;\;\;\;\;{\;t}_{i}\leq g_{i4}\\ 1-\frac{g_{i5}-t_{i}}{g_{i5}-g_{i4}}\;\;{\;g}_{i4}{<t}_{i}{<g}_{i5}\\ 1\;\;\;\;\;\;{\;t}_{i}{>g}_{i5} \end{array}$$

Note: t_i_ is the actual test value of the i-th evaluation index; g_ij_ is the standard value of grade j water quality of the i-th evaluation index; a_ij_ is the subordination degree of the i-th evaluation index to j grade water quality. The fuzzy relation evaluation matrix A can be determined by the membership function established above:

(4) $$A=\left(\begin{array}{ccc} a_{11} & \cdots & a_{15}\\ \vdots & \ddots & \vdots \\ a_{51} & \cdots & a_{55} \end{array}\right)$$

3. Determine weight coefficient matrix

Since different influencing factors have different contributions to water quality, it is necessary to calculate the weight of each factor to make the evaluation model more scientific [19]. Entropy weight method is widely used, because it can effectively take into account the difference degree of each indicator and objectively reflect the importance of each indicator. In this study, the weight coefficient is determined by entropy weight method.

The original data consists of m evaluation objects and n evaluation indicators, which form a T matrix:

(5) $$T=\left(\begin{array}{c} t_{11}\;t_{12}\;\cdots \;t_{1m}\\ \vdots \;\;\vdots \;\;\;\vdots \\ t_{n1}\;t_{n2}\;\cdots \;t_{\mathrm{nm}} \end{array}\right)$$

Use the formula:

$$p_{\mathrm{ij}}=\frac{\underset{j}{\max}\left\{t_{\mathrm{ij}}\right\}-t_{\mathrm{ij}}}{\underset{j}{\max}\left\{t_{\mathrm{ij}}\right\}-\underset{j}{\min}\left\{t_{\mathrm{ij}}\right\}}$$

Standardize the matrix T to obtain the judgment matrix P:

$$P=\left(\begin{array}{c} p_{11}\;p_{12}\;\cdots \;p_{1m}\\ \;\vdots \;\;\;\;\;\vdots \;\;\;\;\;\vdots \\ p_{n1}\;p_{n2}\;\cdots \;p_{\mathrm{nm}} \end{array}\right)$$

(6) $$w_{\mathrm{ei}}=\frac{1-R_{i}}{n-\sum_{i=1}^{n}R_{i}}$$

Note: $R_{i}=\frac{-\sum_{j=1}^{m}y_{\mathrm{ij}}\ln y_{\mathrm{ij}}}{\ln m},y_{\mathrm{ij}}=\frac{1+p_{\mathrm{ij}}}{\sum_{j=1}^{m}\left(1+p_{\mathrm{ij}}\right)}$, obtain matrix W.

4. Establish fuzzy comprehensive evaluation model

Composite the calculation of the fuzzy relation matrix and the weight coefficient matrix:

(7) Q=W×A

The fuzzy relation matrix Q indicates the degree of subordination of water samples to different levels of water quality. The level of the highest degree of subordination is the water quality grade to which the water samples belong.

## 4. Results and Discussion

### 4.1. Analysis of Karst Groundwater Chemical Content Characteristics

Na^+^, K^+^, Ca^2+^, Mg^2+^, SO_4_^2−^, Cl^−^, HCO_3_^−^, and other conventional components account for more than 90% of inorganic content in groundwater, so the conventional components determine the hydrochemical type of groundwater [9]. Due to the content of K^+^ in groundwater in the coal mining area is far lower than that of Na^+^, and the nature of K^+^ is similar to that of Na^+^, thus K^+^ can be unconditionally incorporated into Na^+^ in hydrochemical statistical analysis [26]. According to the production situation of Hengyuan Coal Mine, the sampling time is divided into three stages. The coal mining period before grouting (2007–2013) is stage I, the grouting period (2018–2020) is stage II, and the coal mining period after grouting (2021–2022) is stage III. The hydrochemical indexes of conventional components of karst groundwater samples of Taiyuan Formation in each stage are statistically analyzed. The statistical results are shown in Table 2.

The pH of karst groundwater of Taiyuan Formation in the study area is between 7.12–7.92 (Table 2), which is weakly alkaline water. All samples meet the pH limit range (6.5–8.5) of class III water quality standard specified in China Groundwater Quality Standard (GB/T14848-2017). However, the TDS content of all samples is more than 1000 mg/L, and the average content exceeds 2.4 times of the standard limit value specified by class III water for TDS, with the over-standard rate of 100%, indicating that the content of some ions in karst groundwater is too high, and these ions are likely to affect the karst groundwater quality.

Na^+^+K^+^, Ca^2+^, and Mg^2+^ as the major cation in karst groundwater, have large coefficient of variation (CV > 10%), indicating that they have been disturbed by the outside world [27]. Obviously, the production activities in the mining area (such as coal mining, stratum grouting, etc.) are important factors that affect the change of conventional components in karst groundwater.

**Figure 4:**
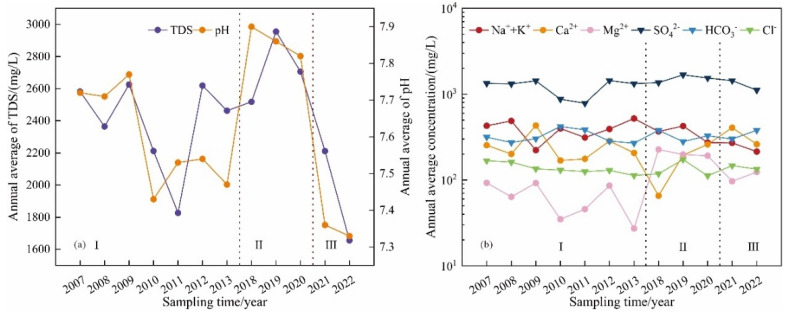
Variation characteristics of chemical indexes of karst groundwater in Taiyuan Formation. (**a**) Evolution of annual average content of TDS and pH, (**b**) Evolution of Annual Average Content of Cations and Anions.

TDS and pH have similar trends (Figure 4a), indicating that the main ion content in karst groundwater of Taiyuan Formation is related to pH to some extent. It can be seen from Figure 4b that the content of cations in karst groundwater changes from high to low as Na^+^+K^+^ > Ca^2+^ > Mg^2+^, the main cation is Na^+^+K^+^, the content of anions changes as SO_4_^2−^ > HCO_3_^−^ > Cl^−^, and the main anion is SO_4_^2−^. The main component of cement is CaO, SiO_2_, Al_2_O_3_, Fe_2_O_3_. Na^+^+K^+^, and SO_4_^2−^ are not the main components of cement. Therefore, the high content of Na^+^+K^+^ and SO_4_^2−^ has no direct relationship with grouting.

Cl^−^ has a high solubility in groundwater, and is not easy to be precipitated, adsorbed, or ingested by bacteria, which can usually be used to explain the chemical evolution of groundwater [28]. The change trend of Cl^−^ content is stable (Figure 4b), and all samples are in line with the standard limit of Cl^−^ content specified by class III water in China Groundwater Quality Standard (GB/T 14848-2017) (Table 2). Considering that the study area is located in the inland of China, and the karst groundwater of Taiyuan Formation is deeply buried, it is difficult to be affected by seawater or polluted by domestic sewage, so Cl^−^ may come from weathering and dissolution of sedimentary rocks or chlorine-containing minerals.

SO_4_^2−^ has the highest ion content in Taiyuan Formation karst groundwater (Figure 4b), with the average content exceeding 5.2 times of class III water quality standard specified in China Groundwater Quality Standard (GB/T14848-2017) (the maximum exceeding standard multiple of SO_4_^2−^ is 13.1 times), and the over-standard rate of SO_4_^2−^ is 100%, which may be related to the dissolution of gypsum or other sulfate sedimentary rocks [29]. In addition, there are thin coal seams in the limestone strata of Taiyuan Formation in the study area, and pyrite is rich in content. The oxidation of pyrite makes a large amount of sulfide insoluble in water enter the groundwater in the form of SO_4_^2−^, which may be another reason for the high content of SO_4_^2−^ in karst groundwater [30].

### 4.2. Fuzzy Comprehensive Evaluation of Karst Groundwater Quality

In this study, the improved fuzzy comprehensive evaluation is adopted, taking the China Groundwater Quality Standard (GB/T14848-2017) as the evaluation standard (Table 1), the evaluation object is all the karst groundwater samples, and TDS, Na^+^, Cl^−^, and SO_4_^2−^ as the evaluation indicators are selected to evaluate the karst groundwater quality of Taiyuan Formation in Hengyuan Coal Mine.

Taking a water sample data in the stage III as an example, the membership degree is calculated according to Formulas (1)–(3), the fuzzy relation matrix A is determined:

$$A=\left(\begin{array}{ccccc} 0 & 0 & 0 & 0 & 1\\ 0 & 0 & 0 & 0.626 & 0.374\\ 0 & 0.022 & 0.978 & 0 & 0\\ 0 & 0 & 0 & 0 & 1 \end{array}\right)\begin{array}{c} \mathrm{TDS}\\ {\mathrm{Na}}^{+}\\ {\mathrm{Cl}}^{-}\\ {\mathrm{SO}}_{4}^{\;2-} \end{array}$$

According to the principle of entropy weight method, the weights of different evaluation factors in fuzzy comprehensive evaluation are calculated by Formula (7):

W = (0.2835,0.2003,0.2267,0.2895)

With Formula (8), the subordination degree of the water sample to each quality grade of groundwater is calculated:

Q = W × A = (0,0.005,0.2217,0.1254,**0.6479**)

According to the principle of maximum membership degree, 0.6479 is the maximum of the five numbers, so the water sample belongs to class V water.

According to the above steps, fuzzy comprehensive evaluation is carried out on other water samples, and the statistical results are shown in Table 3.

In the karst groundwater samples of Taiyuan Formation, Class V water accounts for more than 80%, and the water quality is poor, so it is not suitable for long-term drinking (Table 3). The poor quality of karst groundwater is mainly due to the high content of SO_4_^2−^ and Na^+^+K^+^ in the water. In order to explore the origin of karst groundwater quality in Taiyuan Formation, it is necessary to conduct an in-depth study on the hydrogeochemical process affecting karst groundwater.

### 4.3. Hydrogeochemical Evolution Process

#### 4.3.1. Hydrochemical Characteristics and Evolution

The chemical characteristics of groundwater depend on many factors, such as geological and hydrogeological background, rock weathering, climatic conditions, and human activities [17,31]. These factors interact to produce complex hydrochemical characteristics. Piper diagram is usually used to evaluate the chemical characteristics and evolution process of groundwater [32].

**Figure 5:**
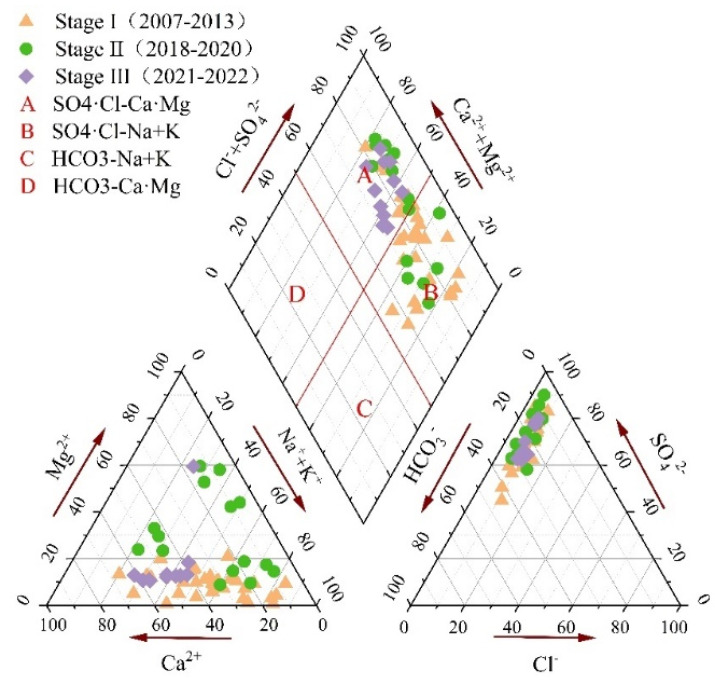
Piper diagram of conventional components of karst groundwater in Taiyuan Formation.

As shown in Piper diagram (Figure 5), all water sample points fall in areas A and B. A total of 43% of the water sample points in stage I are located in area A, 67% of the water sample points in stage II are located in area A, and 100% of the water sample points in stage III are located in area A, indicating that the hydrochemical type of karst groundwater in Taiyuan Formation gradually evolved from SO_4_·Cl-Na+K type to SO_4_ Cl-Ca Mg type.

**Figure 6:**
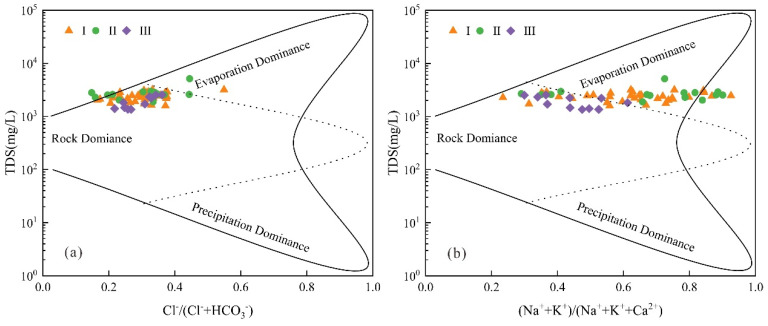
Gibbs diagram of karst groundwater in Taiyuan Formation. (**a**) TDS vs. Cl^−^/(Cl^−^+ HCO_3_^−^), (**b**) TDS vs. (Na^+^+K^+^)/(Na^+^+K^+^+Ca^2+^).

Gibbs diagram can be used to judge the hydrochemical evolution mechanism controlled by atmospheric precipitation, rock weathering, and evaporation concentration [33]. Figure 6a shows that the main ion content of karst groundwater in Taiyuan Formation is dominated by rock weathering. In Figure 6b, part of the water sample points in stage I and stage II of karst groundwater are located outside the dominant evaporation concentration area and the solid line area, indicating that besides rock weathering, evaporation concentration and other hydrochemical processes influent the chemical evolution of karst groundwater. All the water samples in stage III are located in the dominant area of rock weathering (Figure 6b), which shows that the karst groundwater is less affected by evaporation and concentration after grouting. In combination with the smaller the coefficient variation of Cl^−^ in the karst groundwater in stage III (Table 2), it shows that the disturbance intensity of mining activities on karst groundwater is weakened, and the stratum grouting has achieved good results in sealing karst groundwater.

**Figure 7:**
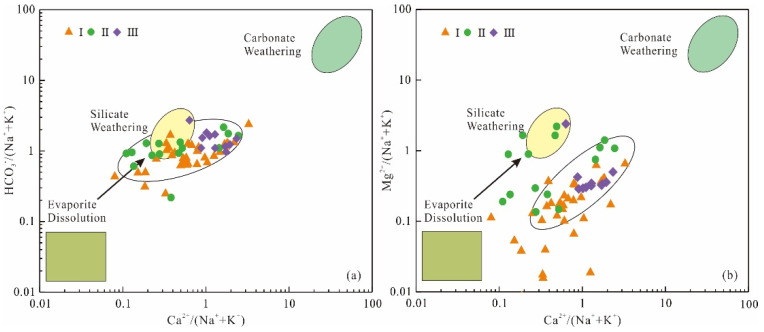
Source distribution of samples by composition ratios. (**a**) HCO_3_^−^/(Na^+^+K^+^) vs. Ca^2+^/(Na^+^+K^+^), (**b**) Mg^2+^/(Na^+^+K^+^) vs. Ca^2+^/(Na^+^+K^+^).

In order to further understand the rock types that play a leading role in the evolution of karst groundwater in the process of rock weathering, the relationship diagrams of HCO_3_^−^/(Na^+^+K^+^) vs. Ca^2+^/(Na^+^+K^+^) and Mg^2+^/(Na^+^+K^+^) vs. Ca^2+^/(Na^+^+K^+^) were drawn. As can be seen from Figure 7a, most of the water sampling points are mainly located in the silicate weathering predominance area, indicating that the hydrochemical evolution of the karst groundwater of Taiyuan Formation in the study area is affected by silicate dissolution. The remaining water samples are located in the dominant area of evaporite dissolution (Figure 7b), indicating that evaporite dissolution is involved in the chemical evolution process of karst groundwater.

#### 4.3.2. Source Analysis of Main Ions in Karst Groundwater

The proportion of main ions produced by weathering and dissolution of rocks is certain [34]. Therefore, the molar ratio of main ions has been widely used to study the mechanism of water-rock interaction and the analysis of ion sources in groundwater [35].

**Figure 8:**
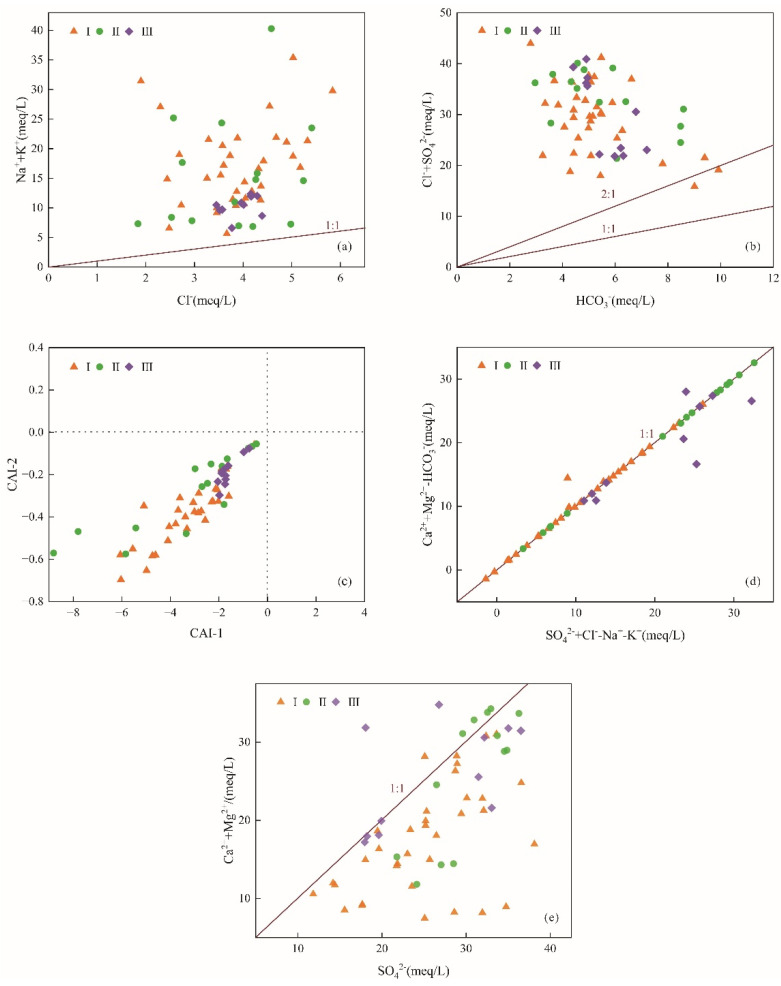
Molar ratio diagram of main ions in karst groundwater. Plots of (**a**): (Na^+^+K^+^) versus Cl^−^; (**b**): (Cl^−^+SO_4_^2−^) versus HCO_3_^−^; (**c**): CAI-2 versus CAI-1; (**d**): (Ca^2+^+Mg^2+^-HCO_3_^−^) versus (SO_4_^2−^+Cl^−^-Na^+^-K^+^); (**e**): Ca^2+^+Mg^2+^ versus SO_4_^2−^.

Rock salt mainly includes halite and sylvite, which is often associated with gypsum as mineral paragenesis. Rock salt dissolution releases equal amounts of Na^+^(K^+^) and Cl^−^ (Equation (8)). Under natural conditions, the value of γ(Na^+^+K^+^)/γ(Cl^−^) in groundwater is about 1 [36]. All sampling points of karst groundwater in Taiyuan Formation are located above γ(Na^+^+K^+^)/γ(Cl^−^) = 1 (Figure 8a), and the average content of Na^+^+K^+^ is 2.74 times that of Cl^−^, indicating that rock salt dissolution is not the only source of Na^+^+K^+^. Na^+^+K^+^ may also come from other processes, including silicate dissolution and cation exchange [37]. Most of the water sample points of karst groundwater fall above the isoline γ(Cl^−^+SO_4_^2−^)/γ(HCO_3_^−^) = 2 (Figure 8b), which indicates that silicate minerals have a certain contribution to Na^+^ content. Combined with the regional stratigraphic lithology and the analysis results of HCO_3_^−^/(Na^+^+K^+^) vs. Ca^2+^/(Na^+^+K^+^) relationship diagram (Figure 7a), it can be concluded that the excessive Na^+^ comes from weathered debris composed of silicate minerals, such as albite (Equation (9)).

(8) $${\mathrm{Halite}:\;\mathrm{Na}(K)\mathrm{Cl}=\mathrm{Na}}^{+}{(K}^{+}{)+\mathrm{Cl}}^{-}$$

(9) $${\mathrm{Albite}:\;\mathrm{Na}}_{2}{\mathrm{Al}}_{2}{\mathrm{Si}}_{6}O_{16}{+2\mathrm{CO}}_{2}{+3H}_{2}{O=2\mathrm{Na}}^{+}{+2\mathrm{HCO}}_{3}{}^{-}{+H}_{4}{\mathrm{Al}}_{2}{\mathrm{SiO}}_{9}{+4\mathrm{SiO}}_{2}$$

Two chlor-alkali indices (CA-1, CA-2) are usually used to indicate the possibility of ion exchange in groundwater, and the greater the absolute value, the stronger the ion exchange effect [38]. The two indices are calculated as follows:

(10) $$\mathrm{CAI}-1=\frac{{\mathrm{Cl}}^{-}-\left({\mathrm{Na}}^{+}{+K}^{+}\right)}{{\mathrm{Cl}}^{-}}$$

(11) $$\mathrm{CAI}-2=\frac{{\mathrm{Cl}}^{-}-\left({\mathrm{Na}}^{+}{+K}^{+}\right)}{{\mathrm{HCO}}_{3}^{-}{+\mathrm{SO}}_{4}^{2-}}$$

If these two indices are positive values, it means that the cation exchange reaction shown in Formula (12) occurs; if they are negative values, it means that the reverse cation exchange reaction occurs, which is shown as Formula (13) [15]. In this study, all water samples fall in the lower left area of Figure 8c, and both indices are negative, indicating that the reverse cation exchange shown in Formula (13) occurs. Ca^2+^ and Mg^2+^ in groundwater are adsorbed by mineral surfaces in surrounding rocks to exchange Na^+^ and K^+^ in rocks. X is an exchange complex, stands for mineral or organic matter.

(12) $${2\mathrm{Na}}^{+}{(K}^{+}{)+\mathrm{Ca}(\mathrm{Mg})X}_{2}{=\mathrm{Ca}}^{2+}{(\mathrm{Mg}}^{2+})+2\mathrm{Na}(K)X$$

(13) $${\mathrm{Ca}}^{2+}{(\mathrm{Mg}}^{2+}{)+2\mathrm{Na}(K)X=\mathrm{Ca}}^{2+}{(\mathrm{Mg}}^{2+}{)X}_{2}{+2\mathrm{Na}}^{+}{(K}^{+})$$

As can be seen from Figure 8d that the ratio of γ(Ca^2+^+Mg^2+^-HCO_3_^−^)/γ(SO_4_^2−^+Cl^−^-Na^+^-K^+^) is close to 1, indicating that SO_4_^2−^ in karst groundwater mainly comes from the dissolution of gypsum [39]. If gypsum is the only dissolved mineral, the molar ratio of γ(Ca^2+^+Mg^2+^):γ(SO_4_^2−^) in groundwater should be 1:1 (Equation (14)) [40]. As shown in Figure 8e, most of the water sample points of karst groundwater fall below the contour line γ(Ca^2+^+Mg^2+^)/γ(SO_4_^2−^) = 1, indicating that except gypsum dissolution, the excess SO_4_^2−^ may come from the oxidation of pyrite (Equation (15)) [41].

(14) $${\mathrm{Gypsum}:\;\mathrm{CaSO}}_{4}{\cdot 2H}_{2}{O=\mathrm{Ca}}^{2+}{+\mathrm{SO}}_{4}{}^{2-}{+2H}_{2}O$$

(15) $${\mathrm{Pyrite}:\;4\mathrm{FeS}}_{2}{+15O}_{2}{+14H}_{2}O=4\mathrm{Fe}{\left(\mathrm{OH}\right)}_{3}\;↓{+8\mathrm{SO}}_{4}{}^{2-}{+16H}^{+}$$

To sum up, the high content of Na^+^+K^+^ in groundwater in Taiyuan Formation is related to cation exchange, and weathering and dissolution of rock salt and silicate. SO_4_^2−^ mainly comes from the dissolution of gypsum and the oxidation of pyrite.

#### 4.3.3. Dissolution/Precipitation Equilibrium of Minerals

Saturation index (SI) is often used to characterize the hydrochemical state and reaction process of minerals relative to groundwater. When a certain mineral SI > 0, the mineral is in a supersaturated state. When SI = 0, the dissolved/precipitated state of this mineral in groundwater is in dynamic equilibrium, while when SI < 0, the mineral is in unsaturated state, and dissolution occurs in groundwater [42]. If the SI value of a mineral is obviously less than 0, it may be a non-reactive mineral. Using PHREEQC software to evaluate the saturation index of minerals in the study area.

**Figure 9:**
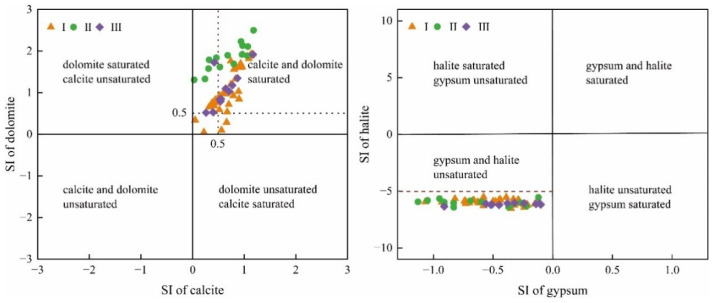
Mineral saturation index of karst groundwater in Taiyuan Formation.

It can be seen from Figure 9 that calcite and dolomite are in supersaturated state, gypsum and halite are in dissolved state all the time, and the dissolved/precipitated state of minerals in each water sample point is basically consistent. The saturation index of calcite and dolomite in most water sampling points is greater than 0.5, indicating that the saturation degree of these two minerals in karst groundwater is always high, and they are easy to precipitate. The saturation index of halite is less than −5, which indicates that karst groundwater has a strong ability to dissolve halite, which also indicates that the dissolution of halite has little effect on the chemical composition of groundwater.

#### 4.3.4. Correlation Analysis

**Figure 10:**
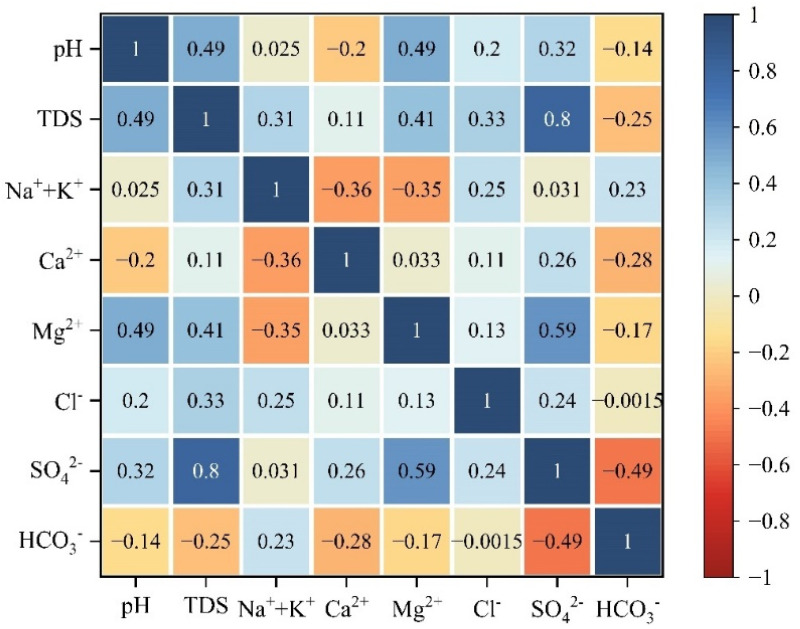
Heat map of correlation coefficient of conventional components of karst groundwater in Taiyuan Formation.

Correlation analysis is often used to analyze the potential sources of ions in hydrochemical evolution, and components from the same source usually have significant correlation [43]. It can be seen from Figure 10 that Na^+^+K^+^ is positively correlated with Cl^−^ and negatively correlated with Ca^2+^ and Mg^2+^, indicating that Na^+^+K^+^ comes from the dissolution of rock salt and cation exchange. This is consistent with the analysis result of the ion ratio analysis diagram (Figure 8a).

SO_4_^2−^ is positively correlated with pH, and the content of pyrite in coal seam is abundant, which indicates that pyrite oxidation has a certain contribution to the content of SO_4_^2−^ in groundwater [44]. The oxidation of pyrite products H^+^, which can accelerate the dissolution of dolomite and calcite in the limestone of Taiyuan Formation, and the dissolution of dolomite and calcite can buffer the acidification of groundwater [45]. This is also a reason why groundwater is weakly alkaline.

SO_4_^2−^ is negatively correlated with HCO_3_^−^. Calcite reacts with H^+^ from pyrite oxidation products releasing Ca^2+^ and HCO_3_^−^. The dissolution of gypsum products a large amount of Ca^2+^, which inhibits the dissolution of calcite [46]. This proves once again that the high content of SO_4_^2−^ in the karst groundwater of Taiyuan Formation is related to the dissolution of gypsum and the oxidation of pyrite.

Mg^2+^ and Ca^2+^ are positively correlated with SO_4_^2−^, which corresponds to the dissolution of sulfate. The mineral saturation index of dolomite and calcite has obvious correlation (Figure 9), indicating that the dissolution of dolomite and calcite is synchronous, so Ca^2+^ and Mg^2+^ will have obvious positive correlation. However, it can be seen from Figure 10 that the correlation coefficient between Ca^2+^ and Mg^2+^ is 0.033, with a weak positive correlation. It is speculated that dedolomitization occurred. This will be demonstrated in principal component analysis.

### 4.4. Main Factors Affecting Hydrogeochemical Evolution Process

**Figure 11:**
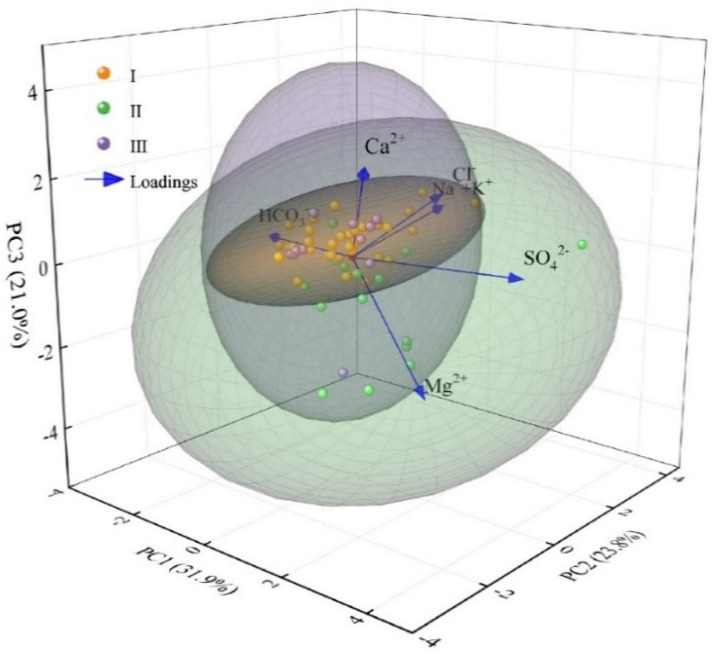
Principal component analysis of conventional components of karst groundwater in Taiyuan Formation.

According to the steps proposed in the section of “Research Methods”, six parameters (Na^+^+K^+^, Ca^2+^, Mg^2+^, Cl^−^, SO_4_^2−^, and HCO_3_^−^) were selected for multivariate statistical processing (PCA). Finally, three principal components (PC1, PC2 and PC3) with eigenvalues greater than 1 were extracted successively through varimax rotation. The initial eigenvalues were 3.871, 1.925, and 1.196, respectively, the variance contribution rates are 45.042%, 21.029%, and 11.613%, respectively, and the cumulative variance contribution rate reached 77.684% (Table 4); it can basically reflect the hydrogeochemical information of karst groundwater of Taiyuan Formation in the study area. In order to analyze the loadings between these hydrochemical parameters more intuitively (the correlation coefficient between the principal components and the analyzed variables is called the loading [47]), the software Origin was used to draw the principal component analysis diagram (default settings in the software Origin were followed for PCA), as shown in Figure 11.

On the principal component PC1, the closer the water sample point is to the right side of PC1 axis, the stronger the positive correlation. SO_4_^2−^, Ca^2+^, Mg^2+^ have higher positive loading value, and HCO_3_^−^ have higher negative loading value (Table 4, Figure 11). Combined with the results of ion proportion analysis and correlation analysis, PC1 is mainly related to sulfate dissolution and pyrite oxidation. The relevant chemical equation is:

$${\mathrm{CaSO}}_{4}{\cdot 2H}_{2}{O=\mathrm{Ca}}^{2+}{+\mathrm{SO}}_{4}{}^{2-}{+2H}_{2}O\;{\mathrm{MgSO}}_{4\;}{=\mathrm{Mg}}^{2+}{+\mathrm{SO}}_{4}{}^{2-}\;{4\mathrm{FeS}}_{2}{+15O}_{2}{+14H}_{2}O=4\mathrm{Fe}{\left(\mathrm{OH}\right)}_{3}↓{+8\mathrm{SO}}_{4}{}^{2-}{+16H}^{+}$$

Similarly, in PC2, Na^+^+K^+^ and Cl^−^ have higher positive loading value, and Ca^2+^ have higher loading value. Based on the previous discussion results, it can be inferred that PC2 is mainly related to cation exchange, dissolution of rock salt and silicate. Considering the different affinities between different cations, Ca^2+^ is usually adsorbed better than Mg^2+^ in most cases [48]. Therefore, it is speculated that Ca^2+^ is the main ion involved in cation exchange. The related chemical equation is:

$${\mathrm{Na}}^{+}{{(K}^{+})}_{\mathrm{clay}}{+\mathrm{Ca}}^{2+}{}_{\mathrm{water}}\to {\mathrm{Na}}^{+}{{(K}^{+})}_{\mathrm{water}}{+\mathrm{Ca}}^{2+}{}_{\mathrm{clay}}\;{\mathrm{Na}(K)\mathrm{Cl}=\mathrm{Na}}^{+}{(K}^{+}{)+\mathrm{Cl}}^{-}\;{\mathrm{Na}}_{2}{\mathrm{Al}}_{2}{\mathrm{Si}}_{6}O_{16}{+2\mathrm{CO}}_{2}{+3H}_{2}{O=2\mathrm{Na}}^{+}{+2\mathrm{HCO}}_{3}{}^{-}{+H}_{4}{\mathrm{Al}}_{2}{\mathrm{SiO}}_{9}{+4\mathrm{SiO}}_{2}$$

The saturation indices of calcite and dolomite are both greater than 0, indicating that these minerals are highly saturated in groundwater and easy to precipitate. A large amount of gypsum dissolution will increase the content of Ca^2+^ in groundwater. When Ca^2+^ reaches a specific level, calcite will be forced to precipitate, which will lead to dedolomitization reaction (Equation (16)) [49,50]. The content of Ca^2+^ in groundwater will decrease and the content of Mg^2+^ will increase. This corresponds to the high positive loading value of Ca^2+^ and higher negative loading value of Mg^2+^ in PC3, indicating that the karst groundwater of Taiyuan Formation in the study area has undergone dedolomitization. If only dedolomitization occurs in groundwater, there will be a significant negative correlation between Ca^2+^ and Mg^2+^. From the contribution rate of PC1 is 31.9% and that of PC3 is 21%, it can be concluded that dedolomitization is not the main reaction path affecting the content of Ca^2+^, and the content of Ca^2+^ is mainly affected by the dissolution of gypsum, which also explains the reason why Ca^2+^ and Mg^2+^ show a weak positive correlation in the correlation analysis (Figure 10). The relevant chemical equation is:

(16) $${\mathrm{Dedolomitization}:\;\mathrm{Ca}}^{2+}+\mathrm{CaMg}{\left({\mathrm{CO}}_{3}\right)}_{2}⇌{\mathrm{Mg}}^{2+}{+2\mathrm{CaCO}}_{3}\;↓$$

### 4.5. Prediction of Hydrochemical Evolution

**Figure 12:**
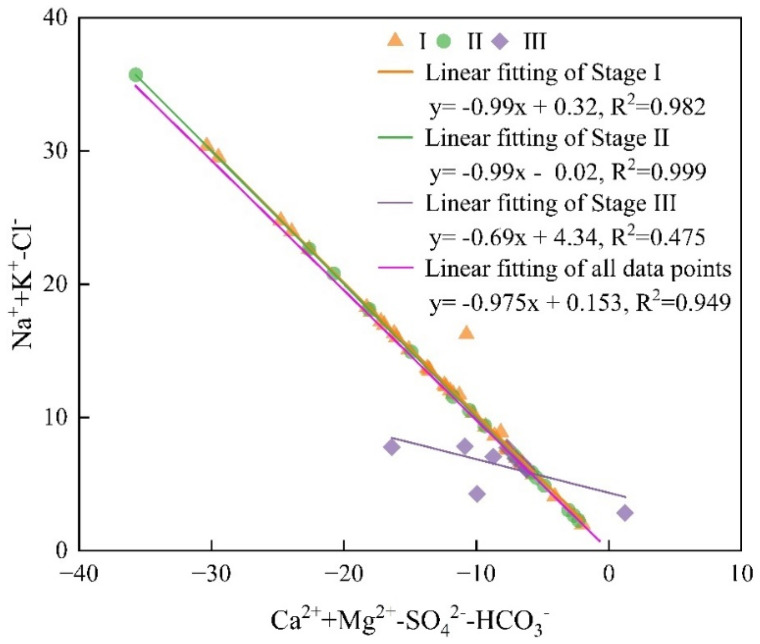
Bivariate diagram of conventional components of karst groundwater in Taiyuan Formation.

The influencing factors of hydrochemical composition of groundwater not only depend on the chemical composition of surrounding rock, but also relate to the degree of ion exchange [9]. In addition to the chlor-alkali index, bivariate diagram ((Ca^2+^+Mg^2+^-SO_4_^2−^-HCO_3_^−^), and (Na^+^+K^+^-Cl^−^)) can be used to judge whether cation exchange occurs. If the slope is close to -1, it indicates that the cation exchange is strong [51]. Through linear fitting (Figure 12), all water samples of karst groundwater in Taiyuan Formation in the study area have good linear relationship, showing a straight line with a slope of −0.975 and R^2^ of 0.949, which is significantly linearly correlated, indicating that the cation exchange of karst groundwater is obvious, and the cation exchange is the key factor to form the hydrochemical characteristics of karst groundwater.

The limestone of Taiyuan Formation in the study area contains thin coal seams, and the roof clay of coal seams is rich in Ca^2+^ and Mg^2+^, which are easy to be dissolved, migrated and transformed. The slopes of the linear fitting curves of the water samples in stage I and stage II are −0.99, R_1_^2^ = 0.982, R_2_^2^ = 0.999, and the cation exchange is strong. The karst groundwater and the surrounding rock have sufficient leaching and ion exchange, and a large amount of Ca^2+^(Mg^2+^) is fully replaced by Na^+^ + K^+^ in the surrounding rock, which is also an important for the high content of Na^+^ + K^+^ in the karst groundwater of Taiyuan Formation in the study area. In stage III, the linear fitting curve is −0.69, R_3_^2^ = 0.475, the cation exchange effect is obviously weakened, and the content of Na^+^+K^+^ decreases.

**Figure 13:**
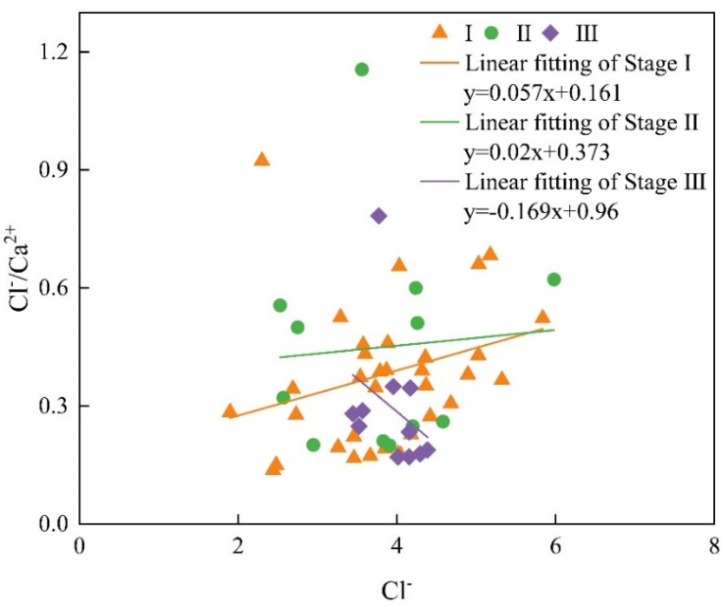
Analysis of karst groundwater hydrodynamic conditions in Taiyuan Formation.

In addition, the degree of ion exchange also depends on the length of groundwater retention time [38]. The relationship between Cl^−^/Ca^2+^ and Cl^−^ can reveal the hydrodynamic conditions of groundwater (Figure 13) [52]. If Cl^−^/Ca^2+^ is inversely proportional to Cl^−^, it indicates that groundwater has good hydrodynamic conditions [26]. The weak interaction between groundwater and surrounding rock reduces the risk of over dissolution. The line fitting slopes of Taiyuan Formation karst groundwater sample points in stage I and stage II are 0.057 and 0.02, respectively, indicating that the groundwater runoff is relatively slow. The slope of the groundwater samples in stage III is −0.169, indicating that the karst groundwater has good hydrodynamic conditions in stage III compared with stage I and stage II. Through analysis, the grouting treatment has effectively blocked the groundwater diversion channel, accelerated the water circulation of karst groundwater, shortened the residence time, and weakened the leaching and ion exchange between karst groundwater and surrounding rock. This is consistent with the analysis result of Gibbs diagram (Figure 5). Although the weak interaction between groundwater and surrounding rock reduces the risk of over dissolution, such fast hydrodynamics may bring the risk that organic contaminants cannot be completely degraded during the transportation, which may be a problem for drinking water production.

Sulfate reduction is one of the common characteristics of water-rock interaction in coal mine groundwater [53]. The deep coal measures stratum is in an anoxic environment, and the marine clay rock on the roof of the coal seam is rich in Na^+^. After cation exchange adsorption with Ca^2+^ in the groundwater, under the action of coal organic matter and anaerobic sulfate-reducing bacteria, the SO_4_^2−^ in the groundwater is reduced to H_2_S (the desulfurization acid effect) [54]. The reaction formula is:

$${\mathrm{Desulphidation}:\;2\mathrm{Na}}^{+}{+\mathrm{Ca}}^{2+}{\mathrm{SO}}_{4}{}^{2-}\to {\mathrm{Ca}}^{2+}{+\mathrm{Na}}^{+}{}_{2}{\mathrm{SO}}_{4}{}^{2-}$$

(17) $${\mathrm{Na}}^{+}{}_{2}{\mathrm{SO}}_{4}{}^{2-}{+2C(\mathrm{organic}\;\mathrm{matter})+2H}_{2}O\overset{\;\mathrm{Anaerobic}\;\mathrm{sulfate}-\mathrm{reducing}\;\mathrm{bacteria}\;}{\to }{2\mathrm{Na}}^{+}{\mathrm{HCO}}_{3}{}^{-}{+H}_{2}S$$

The circulation of Taiyuan Formation karst groundwater is accelerated, the residence time is shortened, the carbon source is continuously supplemented, and the desulfurization acid effect is enhanced, which also explains the phenomenon that TDS and pH of karst groundwater in Taiyuan Formation have a downward trend and the HCO_3_^−^ content has an upward trend in stage III (Figure 4). Based on this, it is predicted that in the future, due to the sealing effect of stratum grouting on karst groundwater, the circulation of karst groundwater in Taiyuan Formation in the study area was focused which led to lower residence times, the cation exchange is weakened, and the desulfurization acid effect is enhance. The karst groundwater of Taiyuan Formation in the study area will evolve from the current SO_4_-Ca·Mg type to HCO_3_-Ca·Mg type.

Desulfurization can reduce the content of SO_4_ in groundwater to a certain extent, which is a positive effect of karst groundwater as drinking water. However, in terms of organic matter degradation, lower residence time might prove risky, and follow-up studies have to verify whether the ecosystem services can still be maintained under these new conditions.

## 5. Conclusions

In this study, the hydrogeochemical evolution characteristics of karst groundwater in Taiyuan Formation of Hengyuan Coal Mine in Huaibei Coalfield, China was studied by means of hydrochemistry and multivariate analysis. The main hydrochemical processes affecting karst groundwater are discussed in depth. The following conclusions can be drawn:

1. The fuzzy comprehensive evaluation results show that the proportion of class V water in karst groundwater in Taiyuan Formation accounts for more than 80%, and the water quality is poor, which is not suitable for long-term drinking. The high content of Na^+^+K^+^ and SO_4_^2−^ in groundwater is the main reason for poor water quality.
2. By using the Piper diagram, ion ratio analysis, mineral saturation index, and correlation analysis, it is concluded that Na^+^+K^+^ mainly comes from cation exchange, dissolution of rock salt and silicate, and SO_4_^2−^ mainly comes from sulfate dissolution and pyrite oxidation.
3. Through principal component analysis, it is concluded that the main factors controlling the chemistry of karst groundwater in Taiyuan Formation are sulfate dissolution, pyrite oxidation, cation exchange, and dedolomitization.
4. Influenced by stratum grouting, the circulation of karst groundwater in Taiyuan Formation in the study area is accelerated, the cation exchange is weakened, and the desulfurization acid effect is enhanced. In the future, the hydrochemical type of karst groundwater will evolve from SO_4_-Ca Mg type to HCO_3_-Ca·Mg type.

## Figures and Tables

**Table 1:** Standard for classification of groundwater quality in China (GB/T 14848-2017).

Grade	Classification	Parameters (Unit: mg/L)			
		TDS	Na^+^	Cl^−^	SO_4_^2−^
I	Excellent, suitable for drinking water	≤300	≤100	≤50	≤50
II	Good, suitable for drinking water	≤500	≤150	≤150	≤150
III	Moderate, suitable for drinking water	≤1000	≤200	≤250	≤250
IV	Poor, suitable for drinking water	≤2000	≤400	≤350	≤350
V	Unsuitable, suitable for drinking water	>2000	>400	>350	>350

**Table 2:** Statistical table of conventional component content of karst groundwater in Taiyuan Formation.

Indexs	Unit	GIII	Stage I (*n* = 35)			Stage II (*n* = 15)			Stage III (*n* = 11)		
			Range	Mean	CV (%)	Range	Mean	CV (%)	Range	Mean	CV (%)
pH	-	6.5–8.5	7.12–7.82	7.60	2.46	7.78–7.92	7.86	0.57	7.12–7.80	7.34	2.91
TDS	mg/L	1000	1591–3186	2384.8	17.59	1891–5145	2726.6	26.83	1358–2552	1908.5	25.04
Na^+^+K^+^	mg/L	200	130–814	394.7	40.87	157–927	355.6	61.59	152–284	240.1	16.64
Ca^2+^	mg/L	-	49.9–461	246	44.08	32.4–392	173.5	74.14	96.2–486	327.1	40.46
Mg^2+^	mg/L	-	5.5–143	63.2	57.28	49.7–367	205.5	47.49	67.8–364	111.5	76.39
Cl^−^	mg/L	250	68.5–207	137.6	23.38	65.4–192	134.5	28.29	122–155	140	8.23
SO_4_^2−^	mg/L	250	568–1830	1215	26.37	774–3278	1528.6	36.06	862–1754	1260.9	28.90
HCO_3_^−^	mg/L	-	170–605	321.4	29.53	181–524	328.9	35.21	269–439	343.9	16.13

Note: GIII: the class III water quality standard in China’s groundwater quality standard (GB/T14848-2017); *n*: the number of samples; CV: the coefficient of variation.

**Table 3:** Statistical results of fuzzy comprehensive evaluation of karst groundwater in Taiyuan Formation.

Indexs	Class I Water	Class II Water	Class III Water	Class IV Water	Class V Water
Stage I	0	0	0	8.57%	91.43%
Stage II	0	0	6.67%	13.33%	80%
Stage III	0	0	0	18.18%	81.82%

**Table 4:** Principal component analysis results of conventional components of karst groundwater in Taiyuan Formation.

Indexs	PC1	PC2	PC3
Na^+^+K^+^	−0.05	0.782	0.118
Ca^2+^	0.316	−0.369	0.637
Mg^2+^	0.397	−0.06	−0.68
Cl^−^	0.21	0.362	0.337
SO_4_^2−^	0.616	0.308	−0.039
HCO_3_^−^	−0.562	0.152	−0.051
Initial eigenvalue	1.912	1.428	1.259
Contribution rate (%)	31.9	23.8	21
Cumulative contribution rate (%)	31.9	55.67	76.66

## Data Availability

The data used to support the findings of this study are included within the article.
